# Evaluating the impact of a community-based cancer awareness roadshow on awareness, attitudes and behaviors

**DOI:** 10.1016/j.ypmed.2016.02.034

**Published:** 2016-06

**Authors:** Samuel G. Smith, Kirstie Osborne, Sophie Tring, Helen George, Emily Power

**Affiliations:** a Centre for Cancer Prevention, Wolfson Institute of Preventive Medicine, Queen Mary University of London, Charterhouse Square, London EC1M 6BQ, UK; b Health Behaviour Research Centre, Department of Epidemiology and Public Health, University College London, Gower Street, London WC1E 6BT, UK; c Cancer Research UK, Angel Building, 407 St John Street, London EC1V 4AD, UK

**Keywords:** Cancer awareness, Health behaviors, Community interventions, Mobile information units, Roadshow, Health communication, Cancer prevention, Early diagnosis, Cancer control

## Abstract

Improving public awareness of cancer and encouraging health behavior change are important aspects of cancer control. We investigated whether a community-based roadshow was an effective way of communicating with the public about cancer and encouraging behavior change. Data were from 1196 people who completed questionnaires at a Cancer Research UK Cancer Awareness Roadshow in 2013. Of these, 511 (43%) completed questionnaires immediately before their visit (pre-visit group) and 685 (57%) completed questionnaires immediately after their visit (post-visit group). Among the post-visit sample, 217 (32%) were retained after two months. Self-reported data were available on risk factor and symptom awareness, help-seeking barriers, use of healthcare services and health behaviors. Compared with the pre-visit sample, the post-visit group had greater awareness of cancer risk factors and was more positive about aspects of help-seeking but awareness of potential symptoms was similar. Most effects were maintained over two months. Intentions to eat more fruit and vegetables and to exercise more were comparable between the groups but more people in the post-visit sample intended to quit smoking. At 2-month follow-up, smoking prevalence had significantly reduced but fruit and vegetable consumption decreased and there was no change to physical activity. User of weight loss services and general practitioner visits were high at follow-up and largely attributed to the Roadshow. The Cancer Research UK Roadshow appears to improve risk factor awareness, promote positive attitudes towards help-seeking and increase smoking cessation. This approach could be a useful building block for additional cancer prevention and control strategies.

## Highlights

• The Roadshow increased cancer awareness and positive attitudes to help-seeking.
• Higher levels of smoking cessation were seen among visitors at follow-up.
• Use of healthcare services was largely attributed to the Roadshow at follow-up.

## 1 Introduction

In 2014, there were a projected 1.6 million new cancer cases and over 0.5 million cancer-related deaths in the US (Siegel et al., 2014). The latest UK data reported over 330,000 new cancer diagnoses in 2011, and 160,000 cancer-related deaths in 2012 (Office for National Statistics, 2012). It is now estimated that 1 in 2 people in the UK will be diagnosed with cancer in their lifetime (Ahmad et al., 2015). On the basis of current known risk factors, approximately one third to one half of these cancers are preventable through primary and secondary prevention efforts (Vineis and Wild, 2014, Parkin, 2011). Furthermore, a substantial proportion of cancer deaths could be avoided if UK 5-year survival rates matched those of comparable countries (Abdel-Rahman et al., 2009, Coleman et al., 2011). The potential to improve outcomes through lifestyle changes and earlier diagnosis is considerable.

There is concern that the UK public are unaware of a number of important risk factors for the development of cancer (Redeker et al., 2009, Marlow et al., 2012). A substantial proportion of the population fail to meet recommendations for cancer prevention behaviors. One third (33%) of men and 45% of women fail to meet aerobic activity guidelines (Lifestyle statistics team, Health and Social Care Information Centre, 2014), 73% of the population do not eat the recommended number of fruits and vegetables (Lifestyle statistics team, Health and Social Care Information Centre, 2014), and 19% continue to smoke (Top Line Findings from the Smoking Toolkit Study, 2014). Public awareness of cancer signs and symptoms is also low (Forbes et al., 2013, Robb et al., 2009), and has been attributed as a cause of delayed help-seeking and late diagnosis (Robb et al., 2009, Quaife et al., 2014), particularly among lower socioeconomic status (SES) groups (Waller et al., 2009). In the UK, attitudes towards help-seeking, such as fears about wasting doctors' time, are poor in comparison with other European countries (Forbes et al., 2013). Population-based initiatives are needed to increase cancer awareness, encourage more positive attitudes towards help-seeking and promote positive behavior change.

Some attempts to improve cancer awareness and encourage behavior change among the population have been successful. The most notable recent example of this in the UK is the national lung ‘Be Clear on Cancer’ campaign. This ran for 8 weeks and aimed to raise awareness of a persistent cough as a sign of lung cancer and encourage earlier presentation to a GP, particularly among lower SES groups, through television, posters, radio and newspaper coverage (Ironmonger et al., 2015). The campaign was associated with improved public awareness and more lung cancer diagnoses. Importantly, there were also positive shifts in diagnostic stage and number of resections performed. Positive changes in awareness and urgent GP referrals were also reported for the bowel ‘Be Clear on Cancer’ campaign, which used a similar approach (Peacock et al., 2013).

These strategies are particularly noteworthy because they occur in opportunistic settings. Public information events, such as roadshows and health fairs, also allow people to receive information opportunistically, where the majority of cancer-related information is accessed (Niederdeppe et al., 2007). As a result they can increase engagement with cancer prevention and early diagnosis strategies (Redwood et al., 2013, Sanchez et al., 2014, Briant et al., 2014). They also use techniques that have low demands on literacy skills and can overcome barriers such as informational avoidance (Smith et al., Epub ahead of print).

There is a substantial appetite among the public to access health information in this way. Cancer Research UK's Cancer Awareness Roadshow currently receives over 60,000 visits each year. The Roadshow is a multi-component community-based initiative and has been running since 2006. The primary aims of the Roadshow are to increase awareness of cancer risk factors and signs and symptoms, and to encourage behavior change related to the primary and secondary prevention of cancer. It is specifically targeted to reach the public in opportunistic settings in lower SES areas. We have previously reported the Roadshow's effectiveness in improving intention to make lifestyle changes and use local health services, particularly among lower SES groups, ethnic minorities, and smokers (Smith et al., 2014). This study reports awareness, attitudinal and behavioral outcomes related to cancer prevention and early diagnosis using data collected from attendees of Roadshow activity across the country between July and October 2013. We hypothesized that Roadshow attendees (post-visit) would have higher awareness of cancer risk factors and signs and symptoms, and be more positive about cancer and help-seeking, compared with the pre-visit sample. We anticipated that these effects would be maintained at 2-month follow-up. We also hypothesized that Roadshow attendees (post-visit) would have higher intentions to perform cancer control behaviors than the pre-visit group and be more likely to have changed their behavior at follow-up.

## 2 Methods

### 2.1 Study design

Randomization of people attending the Roadshow was not possible. Asking visitors to complete a survey about cancer prevention and early diagnosis before they visited the Roadshow would likely bias their experience and any post-visit responses. Therefore, we interviewed separate groups before and after their visit. The pre-visit sample was interviewed immediately before their visit to the Roadshow, while the post-visit sample was interviewed immediately afterwards. Only the post-visit respondents were followed up after 2 months via telephone (2-month sample) so that comparisons could be made with their responses at the post-visit stage. Follow-up of the pre-visit sample was unnecessary as they were to solely act as a comparator to the post-visit sample.

The same survey was administered to the pre-visit, post-visit and 2-month samples, although the post-visit and 2-month samples were asked additional questions about their visit. The survey took approximately 10 minutes to complete and data were collected by a trained interviewer from an external research company. No incentive was given for participation. People were allocated to complete the survey pre- or post-visit based on quotas given to the interviewers at the start of the day. The interviewers were given discretion as to which group visitors would be assigned to, but were requested to keep a balance throughout the day. Baseline data were collected over a period of 50 days from 27 different locations in the North-West and North-East of England and London.

### 2.2 The Cancer Awareness Roadshow

The Cancer Research UK Cancer Awareness Roadshow is an initiative involving mobile information units and specially trained staff who visit lower SES areas across the UK. The aim is to improve cancer awareness and facilitate behavior change. Visitors can speak with a cancer awareness nurse who will answer questions, provide tailored information and signpost to relevant local health services. There are interactive resources on display to help engage visitors, and people are offered the opportunity to have a BMI test and to take away Cancer Research UK, NHS and other leaflets on a range of cancer-related topics. Smokers are also invited to do a carbon monoxide test. The Roadshow and upcoming locations are promoted online and via twitter, regional press and posters in community settings — but most visitors are opportunistic.

### 2.3 Measures

#### 2.3.1 Cancer risk factor awareness

Using items from the Cancer Awareness Measure (CAM), we assessed awareness of cancer risk factors in the pre-visit, post-visit and 2-month questionnaires (Stubbings et al., 2009). To limit respondent burden, only six of the original 11 items were used here. These items were picked as they were more closely related to lifestyle. Respondents were asked to respond ‘yes’, ‘no’, or ‘not sure’ to the list of items when asked, ‘Do you think each of the following can increase a person’s risk of getting cancer?’. Examples included ‘getting sunburnt’ and ‘drinking alcohol’. Items were summed with each ‘yes’ response allocated a single point. Higher scores indicated greater awareness. The scale was reliable (Cronbach's alpha = 0.75).

#### 2.3.2 Cancer symptom awareness

Using items from the CAM (Stubbings et al., 2009), respondents indicated ‘yes’, ‘no’ or ‘don't know’ when asked, ‘Do you think each of the following could be signs of cancer?’ Respondents were given nine signs, such as ‘an unexplained lump or swelling’ or ‘a change in the appearance of a mole’. Items were summed with each ‘yes’ response allocated a single point. Higher scores indicated greater awareness. The measure was used in the pre-visit post-visit and 2-month questionnaires. The scale was reliable (Cronbach's alpha = 0.82).

#### 2.3.3 Attitudes towards cancer and help-seeking

Attitudes towards cancer were assessed at all time points using the following items: ‘I believe there is nothing people can do to reduce their chances of developing cancer’ (Niederdeppe and Levy, 2007), and ‘I believe that if cancer is diagnosed early it is more likely to be treatable’. Responses were dichotomized to reflect ‘disagree’ (strongly disagree, tend to disagree) and ‘agree’ (strongly agree, tend to agree). Attitudes towards help-seeking were assessed using the following statement and responses: ‘…could you say if any of these might put you off going to the doctor?’ (Stubbings et al., 2009). The options were: ‘I would be worried about wasting the doctor’s time’, ‘I would be worried about what the doctor might find’, and ‘I wouldn't feel confident talking about my symptom with the doctor’. Responses were ‘yes’, ‘no’ and ‘I don't know’.

#### 2.3.4 Cancer control intentions and behaviors

Intentions to eat more fruit and vegetables and participate in more physical activity were asked in the post-visit questionnaire using the items, ‘Over the next month, do you plan on (eating fruit and vegetables any more or less than you do now?), (being physically active any more or less than you are now?)’. Response options were dichotomized as intentions to do more of the behavior (much more, a little more) and less of the behavior or the same amount (much less, a little less, no more or less). Smokers' intention to quit was asked in the post-visit questionnaire using the item: ‘Which of the following statements best applies to you?’ (‘I do not plan to stop smoking in the next 5 years’, ‘I plan to stop smoking in the next month/6 months/5 years’) (Hall et al., 2003). Responses were dichotomized to reflect intention to quit smoking in the next month versus no intention to quit in the next month.

Smoking status was assessed in the pre-visit, post-visit and 2-month questionnaire with the item, ‘do you smoke?’, and ‘if yes, how many cigarettes per day or per week’. Respondents were classified as smokers at any level of smoking. Fruit and vegetable consumption was assessed in the post-visit and 2-month questionnaire with the item, ‘Thinking about last week, how many portions of fruits and vegetables did you eat each day on average?’ (Redeker et al., 2009). Responses ranged from ‘none’ to ‘five or more’, and respondents were classified as eating < 5 a day or ≥ 5 a day. Physical activity was assessed in the post-visit and 2-month questionnaire using the item, ‘Thinking about last week, how many times a week did you do physical activity, at least moderately, for 30 minutes or more?’ Examples of moderate activity were provided (Redeker et al., 2009). Responses ranged from ‘less than once’ to ‘7 times or more’. Respondents were classified as exercising < 5 times a week or ≥ 5 times a week.

Intention to use healthcare services was asked in the post-visit group only. This included the items, ‘As a result of your visit to the Roadshow, do you intend to make an appointment with your GP/access weight loss services’, ‘yes’ or ‘no’. At 2-month follow-up, the post-visit group were asked, ‘Since your visit to the Roadshow, have you visited your GP about an unusual or persistent change to your body / have you accessed any weight loss services’. Those reporting ‘yes' were asked whether the Roadshow influenced their decision to use the service.

#### 2.3.5 Respondent characteristics

Gender, age, employment, housing status, and location of Roadshow were recorded.

### 2.4 Analyses

Differences between the groups on respondent characteristics and study location were analyzed using the chi-square statistic. The proportion of people in the post-visit group who were retained at two months was described using percentages, and differences between groups were compared with chi-square statistic. Reasons for study drop-out were not recorded. All multivariable analyses were adjusted for age, gender, employment, and study location. Differences were expected between the pre- and post-visit samples on awareness (of risk factors and signs/symptoms) and these outcomes were compared using Analysis of Covariance (ANCOVA). To ascertain if any effects were maintained, the post-visit and 2-month follow-up groups were compared using repeated-measures ANCOVA. A non-significant difference in the repeated measures analyses suggested that effects of the Roadshow were maintained between baseline and 2-month follow-up. Partial-eta (ηρ^2^) effect sizes are reported for all ANCOVA analyses. The individual awareness items were described, but not compared statistically to avoid multiple comparisons.

Differences were expected between the pre- and post-visit samples on attitudes towards help-seeking, and these data were described and analyzed using logistic regression. Analyses were repeated comparing the post-visit and 2-month follow-up groups using McNemar's test to ascertain if effects persisted. Differences in the health behavior intention items were expected between the pre- and post-visit samples and comparisons were made using logistic regression. Differences for behavioral outcomes were expected between the post-visit and 2-month follow-up groups and tested using McNemar's test. All McNemar's analyses were repeated using the more conservative ‘exact test’ statistic. Significance was set at p < .05 and analyses were performed in SPSS version 22 and STATA version 13.

## 3 Results

### 3.1 Study sample

Overall, 1196 people completed questionnaires on the day of the Roadshow, with 511 completing them before their visit (pre-visit) and 685 completing them after their visit (post-visit) (Table 1). The pre- and post-visit respondents were mainly female (63.5%), employed (46.6%), and either rented (45.7%) or owned their own home (46.7%). The mean age was 47.9 years (SD = 17.1). Compared with the pre-visit sample, the post-visit group were more likely to be male (p < .05), were less likely to rent their home (p < .05), and were less likely to be recruited in the North-West of England (p < .05 for overall comparison of all groups).

Two hundred and seventeen people (32%) were retained from the post-visit sample at 2-months. Retention rates were similar across age, gender, employment, and housing status (data not shown), but were higher among those recruited in the North-East of England (37%) compared with those recruited in the North-West of England (30%) and London (27%). This difference was not statistically significant (p = 0.07).

### 3.2 Symptom and risk factor awareness

The overall sample combining pre- and post-visit respondents were aware of an average of 7.5 (SD = 2.1) out of the 9 cancer symptoms and 4.7 (SD = 1.6) out of 6 cancer risk factors (Table 2). The post-visit group were able to recognize an average of 4.8 out of 6 cancer risk factors, an additional 0.3 compared with the pre-visit group (M = 4.5, SD = 1.7). In multivariable analysis, the difference between the pre- and post-visit group was significant (p < 0.001; ηρ^2^ = 0.014). The differences in the individual risk factors demonstrated particularly strong effects in favor of the Roadshow for ‘physical activity’ (9.8% difference), ‘fruit and vegetable’ intake (9.1% difference) and alcohol (7.6% difference) (Table 2). The mean number of risk factors correctly recognized at 2-month follow-up suggested the effect of the Roadshow was maintained at follow-up (M = 4.9; SD = 1.4), and this was confirmed in multivariable analyses comparing post-visit and 2-month scores (p > .05; ηρ^2^ = .000).

Pre- and post-visit respondents were aware of an average of 7.4 (SD = 2.1) and 7.5 (SD = 2.1) out of 9 cancer symptoms, respectively. The difference between pre-and post-visit respondents was not significant in multivariable analysis (p > .05; ηρ^2^ = .001). Observing the individual items (Table 2), there were no consistent differences between the pre- and post-visit samples. The mean number of symptoms correctly recognized at 2-month follow-up was 7.6 (SD = 2.1) out of 9 (Table 2).

### 3.3 Attitudes towards cancer and help seeking

The post-visit group were less worried about wasting the doctors time than the pre-visit group (20.4% vs. 23.1%; OR = 1.66, 95% CI = 1.28–2.14, p < 0.001), less likely to report a lack of confidence in talking about a symptom (12.6% vs. 17.9%; OR = 1.54, 95% CI = 1.10–2.15, p = 0.011), less likely to be worried about what the doctor might find (27.8% vs. 38.1%; OR = 0.60, 95% CI, 0.47–0.87, p < 0.001), and less likely to believe that there was nothing people can do to reduce their risk of cancer (10% vs. 15.2%; OR = 1.68, 95% CI = 1.16–2.42) (Table 3). There were no significant differences between pre and post-visit groups for ‘If cancer is diagnosed early, it is more likely to be treatable’ (p > .05). The levels of agreement with most items at 2-month follow-up were comparable with the post-visit group (ps > .05) (Table 3), suggesting these positive attitudes were maintained over time. However, the 2-month group were more likely to agree that they were worried about wasting the doctor's time (34.6%) than the post-visit group (20.6%; p < .001).

### 3.4 Cancer control behavioral intentions and behaviors

Among all attendees, 28.3% reported smoking, 33.0% reported eating more than five portions of fruit and vegetables per day, and 36.8% reported exercising five or more times per week (Table 4). As expected, the pre- and post-visit groups were comparable in multivariable analysis with regard to smoking status (p > .05) and fruit and vegetable consumption (p > .05). However, the post-visit group were unexpectedly more likely to report exercising 5 or more days per week (pre-visit = 32.9%, post-visit = 39.6%; OR = 1.42, 95% CI, 1.10–1.82, p = 0.006).

Among the smokers at pre-visit, approximately two thirds (62.6%) were planning to quit in the next month, and the post-visit group were more likely to indicate this (53.5% vs. 68.5%; OR: 1.74, 95% CI, 1.07–2.81, p = 0.024) (Table 4). Approximately one third of attendees indicated they were intending to eat more portions of fruit and vegetables in the next month (33.9%) and be more physically active (29.4%), but there were no differences in these intentions between the pre and post-visit groups (ps > .05).

Reportedly as a result of the Roadshow, 33.6% of the post-visit group intended to book an appointment with their GP to discuss a bodily sign or symptom and 13.5% intended to access a weight loss service. Approximately one quarter (24.1%) of all those followed up at 2-months had visited their GP, and most (64.0%) attributed this to the Roadshow. People had also accessed weight loss clinics since the Roadshow (15.2%) and 69.7% attributed this to the Roadshow.

Within-subject comparisons were undertaken comparing self-reported preventive health behaviors within the post-visit respondents who were followed up at 2-months. Smoking prevalence was 26.3% in the post-visit sample and 23.0% after 2-month follow-up (p = 0.035).^1^ Nine (15.8%) of the post-visit attendees who were smokers had quit at follow-up, and 2 (1.3%) previous non-smokers reported smoking at follow-up. The proportion of people who ate five or more portions of fruit and vegetables per week was 37.7% post-visit and 26.8% at 2-month follow-up (p = 0.002). Among those who ate five or more portions post visit, 43 (55.1%) were no longer at this threshold at 2 months. Among those who did not eat five or more portions post-visit, 19 (14.7%) reported eating five or more portions at 2 months. The proportion of respondents who were physically active 5 or more times per week was 49.1% post-visit and 50.9% at 2-month follow-up (p > .05).

## 4 Discussion

In this community-based sample, there was evidence to suggest that following a visit to the Cancer Research UK Cancer Awareness Roadshow, people were more aware of cancer risk factors and felt more positive about cancer and early detection behaviors. Also, a number of these effects were maintained after two month follow-up. Results also suggest the Roadshow may have had an effect on smoking behavior and healthcare service use. Targeted community events based in lower SES areas may be a useful addition to efforts to reduce inequalities in cancer prevention and control.

Awareness of cancer risk factors was similar to national estimates (Robb et al., 2009). There was good awareness that alcohol consumption, overweight, and smoking are linked to the development of cancer. Despite this, people interviewed after their visit were more aware of cancer risk factors and this was largely maintained after two months. The size of this effect is equivalent to one visitor in every three learning a new cancer risk factor. When considering that the Roadshow attracts over 60,000 visitors per year, this would translate to 20,000 people being aware of one more cancer risk factor following their visit. The Cancer Research UK Roadshow should therefore be considered a useful tool for disseminating health information to lower SES communities who may not readily seek it (Barbour et al., 2012, Sweeny et al., 2010, Miles et al., 2008, Brashers et al., 2002, Case et al., 2005). Additional efforts may be required to ensure awareness of factors such as diet and physical activity are promoted, as they were the least well recognized in this sample.

Also in line with national estimates (Robb et al., 2009), respondents recognized most potential cancer symptoms. Awareness was particularly high for ‘a lump or swelling’, ‘a changing mole’, and ‘altered bowel/bladder habits’, although ‘a sore that does not heal’ was not recognized by approximately 30% of attendees. Unfortunately, awareness was unaffected by Roadshow attendance. This may be because of ‘ceiling effects’, but could also be explained by the Roadshow placing more importance of acting on persistent or unusual bodily change, without necessarily outlining specific symptoms.

Intention to quit smoking was higher after a Roadshow visit, and smoking behavior may also have been affected, with 16% of smokers reported to have quit in the two month period after their visit. Considering that small reductions in smoking prevalence can have large population level benefits, this is worthy of further investigation. Specifically, randomized studies are needed using objective outcome measures, such as carbon monoxide monitors and saliva cotinine, and with a longer follow-up.

Despite these positive results, among respondents followed up at two months we noted a reduction in fruit and vegetable consumption. The reasons for this are unclear but we hypothesize that people may have been reluctant to accurately report low consumption in the baseline face-to-face interview. It is also possible that the Roadshow made visitors feel more optimistic about their current behaviors, leading to unrealistic estimates of consumption. Risk compensation is a further explanation (Wilde, 1982). For example, if one risk factor is reduced (e.g. smoking cessation), people may feel better able to tolerate risk exposure from another behavior. Finally, we observed no differences in physical activity. This suggests further support is needed to translate increases in awareness into behavior change. This could take the form of additional follow-up with visitors and through further encouraging them to access support through local services. Strategies to help people track their progress towards goals for specific lifestyle factors may also support sustained positive behavior change (Olander et al., 2013, Michie et al., 2009).

Barriers to symptomatic presentation were reported by a large proportion of the sample. The Roadshow reduced fears about wasting the doctor's time, but this finding had reversed at follow-up. The differences between the pre- and post-visit groups suggested that the Roadshow may reassure people about what the GP might find when presenting with a symptom. This finding was sustained at two months. Confidence talking to the GP about symptoms was also higher among the post-visit group. It could be that attendees were reassured and more confident about seeing the GP after visiting the Roadshow but in turn, less inclined to ‘make a fuss’. This finding needs further exploration. Levels of fatalism were lower among the post-visit group, but this was not maintained.

There is clearly a need for publicly acceptable, consistent and repeated messages about cancer prevention and early diagnosis. This is particularly true for groups from lower SES backgrounds who are more likely to have lower levels of literacy, reducing their capacity to engage with written cancer information and interventions. Health fairs and roadshows may not be sufficient to completely eradicate socioeconomic inequalities in cancer but the preliminary benefits of these activities could serve as ‘building blocks’ for targeted follow-up cancer control messages. The cost-effectiveness of such an approach would need to be investigated.

This study had limitations. We used a pre-post design rather than a randomized trial. We are therefore unable to rule out the possibility that our findings were a result of differences between study groups despite controlling for key demographic factors in all analyses. While individual randomization would have been impossible in the current setting, one alternative would be to undertake a cluster-randomized trial comparing the Roadshow with areas not exposed to the intervention. There was a significant drop-out rate between the post-visit and 2-month samples. Although retention was equal across most socio-demographic variables, we cannot rule out the possibility of bias. We have previously reported high levels of attendance at the Roadshow among lower SES individuals (Smith et al., 2014), however, this was not reflected in the sample reported here, suggesting a degree of response bias. Similarly, while the Roadshow aimed to engage ‘hard to reach’ groups, it is possible that a degree of self-selection bias occurred whereby only individuals interested in cancer information visited and completed a questionnaire. Time-constraints prevented us from using open-ended symptom awareness and cancer risk questions, and awareness is likely to have been lower if they were used. Our measures of attitudes and health behaviors were limited to single self-reported items.

## 5 Conclusion

In conclusion, differences between people before and after their visit to the Cancer Research UK Roadshow suggest that it may have increased awareness of cancer risk factors, improved public attitudes towards cancer help-seeking, and encouraged smoking cessation among visitors. Initiatives such as these are particularly important at they can be delivered in an opportunistic community setting, enabling cancer information to be disseminated to hard to reach groups without the need for active information-seeking. Further efforts are needed to ensure that increases in awareness translate into health behavior changes and that these are maintained in the long-term. Community-based roadshows may be best seen as the ‘building blocks’ for additional cancer prevention and control strategies.

## Ethics approval

Cancer Research UK is a Market Research Society company partner and all research is carried out according to the MRS Code of Conduct. This study used anonymized records and datasets available from the Cancer Awareness Roadshow team at Cancer Research UK who had already acquired appropriate permissions from Roadshow visitors.

## Conflict of interest

Smith was funded by Cancer Research UK as an academic advisor on this project. The work was initiated by Cancer Research UK, analyzed by Smith and interpreted and verified by all authors. Osborne, Tring, George and Power are employed by Cancer Research UK and Power and Osborne have honorary research contracts at UCL.

## Transparency Document

Transparency document.

## Figures and Tables

**Table 1:** Sample characteristics across study groups (2013, UK).

	Overall (n = 1196)	Pre-visit (n = 511)	Post-visit (n = 685)	2-Month sample (n = 217)
*Gender*				
Male	437 (36.5)	166 (32.5)	271 (39.6)	77 (35.5)
Female	759 (63.5)	345 (67.5)	414 (60.4)	140 (64.5)

*Age*				
16–30	244 (20.5)	107 (21.1)	137 (20.1)	47 (21.7)
31–45	273 (22.9)	120 (23.6)	153 (22.4)	42 (19.4)
46–60	340 (28.6)	143 (28.1)	197 (28.9)	61 (28.1)
61–75	281 (23.6)	121 (23.8)	160 (23.5)	61 (28.1)
75 +	52 (4.4)	17 (3.3)	35 (5.1)	6 (2.8)

*Employment*				
Employed	554 (46.6)	244 (47.9)	310 (45.6)	97 (44.7)
Unemployed	116 (9.8)	47 (9.2)	69 (10.1)	20 (9.2)
Other	519 (43.7)	218 (42.8)	301 (44.3)	100 (46.1)

*Housing status*				
Own	559 (46.7)	244 (47.7)	315 (46.0)	110 (50.7)
Rent	547 (45.7)	241 (47.2)	306 (44.7)	88 (40.6)
Other	90 (7.5)	26 (5.1)	64 (9.3)	19 (8.8)

*Location*				
North-west	546 (45.7)	257 (50.3)	289 (42.2)	86 (39.6)
North-east	422 (35.3)	169 (33.1)	253 (36.9)	93 (42.9)
London	228 (19.1)	85 (16.6)	143 (20.9)	38 (17.5)

**Table 2:** Cancer risk factor and symptom awareness (2013, UK).

	Overall (%)	Pre-visit (%)	Post-visit (%)	2-Month (%)
*Risk factor*				
Smoking	95.6	93.3	97.2	95.9
Fruit and vegetables	58.9	53.7	62.8	62.2
Overweight	75.6	72.4	78.0	78.8
Alcohol	77.4	73.0	80.6	85.3
Physical activity	63.1	57.5	67.3	68.2
Sunburn	95.5	95.9	95.2	95.4
Total score (mean, SD)	4.7 (1.6)	4.5 (1.7)	4.8 (1.5)	4.9 (1.4)

*Symptom*				
Lump or swelling	91.9	91.8	92.1	89.9
Persistent unexplained pain	74.3	71.8	76.1	74.7
Unexplained bleeding	86.0	86.1	85.8	90.3
Persistent cough	84.7	84.9	84.5	87.1
Change in bowel/bladder habits	87.7	88.1	87.4	89.9
Persistent difficulty swallowing	74.6	73.5	75.5	80.6
Change in the appearance of a mole	92.4	94.3	90.9	91.7
A sore that does not heal	71.0	70.4	71.4	74.2
Unexplained weight loss	84.1	82.8	85.1	83.4
Total score (mean, SD)	7.5 (2.1)	7.4 (2.1)	7.5 (2.1)	7.6 (2.1)

Note: due to small number of ‘don't know’ responses, the n for the total risk score was: pre-visit (n = 502), post-visit (n = 674), 2-month (n = 217). The n for the total symptom score was: pre-visit (n = 502), post-visit (n = 670), 2-month (n = 217).

**Table 3:** Differences in attitudes towards cancer and help-seeking across study groups (2013, UK).

	% ‘yes’ or ‘agree’			
Overall	Pre-visit	Post-visit	2-month
1. Worried wasting doctor's time (n = 1186)	21.6	23.1	20.4	34.6
2. Worried what doctor might find (n = 1169)	32.2	38.1	27.8	28.6
3. Not confident talking about symptom (n = 1163)	14.9	17.9	12.6	12.3
4. Nothing people can do to reduce chances (n = 1196)	12.2	15.2	10.0	13.5
5. Cancer diagnosed early is more treatable (n = 1196)	94.2	94.6	93.9	95.8

Note: n for question 1 (pre = 506, post = 680, 2-month = 211); question 2 (pre = 499, post = 670, 2-month = 213); question 3 (pre = 491, post = 672, 2-month = 212); question 4 (pre = 487, post = 661, n = 215); question 5 (pre = 464, post = 651, 2-month = 213). Differences are due to small number of ‘don't know’ responses.

**Table 4:** Proportion of respondents reporting intention to change behavior and health behavior outcomes (2013, UK).

	Overall (n, %)	Pre-visit (n, %)	Post-visit (n, %)	2 months (n, %)
*Intention*				
Smoking (to quit)	206 (62.6)	69 (53.5)	137 (68.5)	–
Fruit and vegetables (more)	405 (33.9)	171 (33.5)	234 (34.3)	–
Physical activity (more)	350 (29.4)	160 (31.4)	190 (27.9)	–

*Self-reported behavior*				
Smoking (yes)	339 (28.3)	134 (26.2)	205 (29.9)	50 (23.0)
Fruit and vegetables (five +)	392 (33.0)	173 (34.1)	219 (32.2)	56 (26.8)
Physical activity (five +)	436 (36.8)	166 (32.9)	270 (39.6)	109 (50.9)

Note: n for smoking intention (pre-visit = 129, post-visit = 200); n for fruit and vegetable intention (pre-visit = 511, post-visit = 683); n for physical activity intention (pre-visit = 510, post-visit = 680); n for smoking behavior (pre-visit = 511, post-visit = 685); n for fruit and vegetable intention (pre-visit = 507, post-visit = 680); n for physical activity behavior (pre-visit = 504, post-visit = 681).

## Footnotes

1 When using a more conservative ‘exact test’ the p-value for this relationship is p = 0.065. The significance of the remaining analyses performed with McNemar's Test does not change (fruit and vegetables: p = 0.003; exercise: p = 0.724).
